# *Annona muricata*: Comprehensive Review on the Ethnomedicinal, Phytochemistry, and Pharmacological Aspects Focusing on Antidiabetic Properties

**DOI:** 10.3390/life13020353

**Published:** 2023-01-28

**Authors:** Siti Norliyana Zubaidi, Hidayah Mohd Nani, Mohd Saleh Ahmad Kamal, Taha Abdul Qayyum, Syahida Maarof, Adlin Afzan, Norazlan Mohmad Misnan, Hamizah Shahirah Hamezah, Syarul Nataqain Baharum, Ahmed Mediani

**Affiliations:** 1 Institute of Systems Biology (INBIOSIS), Universiti Kebangsaan Malaysia, Bangi 43600, Selangor, Malaysia; 2 Faculty of Dentistry, Lincoln University College, Petaling Jaya 47301, Selangor, Malaysia; 3 Science and Food Technology Research Centre, Malaysian Agricultural Research and Development Institute, MARDI, Serdang 43400, Selangor, Malaysia; 4 Herbal Medicine Research Centre, Institute for Medical Research, National Institutes of Health, Ministry of Health Malaysia, Shah Alam 40170, Selangor, Malaysia

**Keywords:** *A. muricata*, diabetes, anti-diabetic effect, pharmacology, *Annona* species, metabolite changes

## Abstract

Plants have played an important role over the centuries in providing products that have been used to help combat ailments and diseases. Many products originating from fresh, dried-plant materials, or extracts are utilized as community remedies in traditional practices or even in modern medicine. The Annonaceae family contains different types of bioactive chemical properties, such as alkaloids, acetogenins, flavonoids, terpenes, and essential oil, meaning the plants in this family are potential therapeutic agents. Belonging to the Annonaceae family, *Annona muricata* Linn. has recently attracted the attention of scientists for its medicinal value. It has been utilized as a medicinal remedy since ancient times to treat and improve various diseases, for example, diabetes mellitus, hypertension, cancer, and bacterial infections. This review, therefore, highlights the important characteristic and therapeutic effect of *A. muricata* along with future perspectives on its hypoglycemic effect. The most-common name is soursop, referring to its sour and sweet flavors, while in Malaysia, this tree is commonly called ‘durian belanda’. Furthermore, *A. muricata* contains a high content of phenolic compounds in the roots and leaves. In vitro and in vivo studies have shown that *A. muricata* has the pharmacological effects of anti-cancer, anti-microbial, antioxidant, anti-ulcer, anti-diabetic, anti-hypertensive, and wound healing. With regard to its anti-diabetic effect, mechanisms of inhibiting glucose absorption via α-glucosidase and α-amylase activity inhibition, increasing glucose tolerance and glucose uptake by peripheral tissues, and stimulating insulin release or acting like insulin were deeply discussed. There is still a significant research gap, and future studies are required to conduct detailed investigations and gain a better molecular understanding of *A. muricata*’s anti-diabetic potential, especially by using the metabolomics approach.

## 1. Introduction

Plants have had various significant benefits for thousands of years worldwide, discovered based on traditional knowledge. Even now, plants continue to contribute to new remedies for human beings [1]. *Annona muricata* Linn. (Annonaceae) has been utilized as a medicinal remedy for many years, attracting many scientists to investigate this plant. *A. muricata* L. is a lowland tropical fruit-bearing tree of the Annonaceae family. Graviola, soursop, durian belanda, and guanabana are the popular Malaysian local names for *A. muricata*. Annona’s genus name might be derived from the Latin word ‘anon,’ which means ‘yearly produce’. It could be elaborated as “the fruit production habits of the numerous species in this genus”. On the other hand, Britton and Wilson (1924) suggested that this genus name was derived from ‘Hanon’, which means “the aboriginal name of tropical America tree which is likely to be Santo Domingo”. The name soursop refers to the sour and sweet flavors of the fruit [2]. Many studies reported the therapeutic effects of *A. muricata,* such as anti-tumor, anti-helminth, anti-fungal, anti-bacterial, hypotensive, anti-viral, and anti-inflammatory effects [3,4,5,6,7]. Various parts of *A. muricata*, such as leaves and bark, have been used for medicinal purposes. Over 200 chemical compounds have been discovered and extracted, including phenolics, acetogenins, and alkaloids [8]. Due to its medicinal and pharmacological effects, this plant is considered a potential alternative treatment for diabetes mellitus (DM), hypertension, cancer, and bacterial infections [9,10]. In addition, it is also cheap, can be easily accessed, and has environmental friendliness compared to current commercialized medications, which is a good package to be considered for new potential medications [11]. 

The use of *A. muricata* extracts as a therapeutic drug in managing diabetes mellitus (DM) has yet to be fully explored. DM is a condition in which the body produces insufficient insulin or does not respond to it, causing a relatively high amount of glucose within the blood vessel. Lack of glucose in the tissue has led to an increase in thirst, frequent urination, and feelings of hunger [12]. Patients with DM also tend to lose weight due to insufficient energy. There were 382 million cases of diabetes worldwide in 2013, but that figure is projected to balloon to 592 million by the year 2035. The majority of diabetics and the highest growth in new cases of diabetes during the next 22 years will be found in low- and middle-income countries [13]. Long-term usage of diabetic medication may lead to certain side effects and complications. Hence, it is crucial to develop a medication from natural sources with fewer side effects [14]. Despite the development of new synthetic drugs and their scientific confirmation, research in the scientific community globally continues to explore the anti-diabetic properties of natural products without unwanted effects, whether in the unprocessed or formulated form [15]. Since DM is a global disease expected to rise throughout the year, it has become imperative to research this topic. Other than focusing on its anti-diabetic effect, this review also covers the traditional application of *A. muricata*, its medicinal usage, pharmacological effects, and phytochemical constituents to relate and propose its mechanism of action in DM disease. 

The Annonaceae family has about 2300 species and 130 genera that most likely originated from Northern, Central, and South America [2]. A review of nine *Annona* species found that five of them, *A. muricata*, *Annona reticulata*, *Annona cherimola*, *Annona squamosa*, and *Annona macrophyllata,* possess anti-diabetic properties [16]. Another study on well-known *Annona* species revealed that the roots of *A. reticulata* (82.08 ± 0.74 mg gallic acid equivalent, GAE/g DW) have the highest amount of total phenolic content (TPC), followed by the roots of *A. muricata* (73.10 ± 0.72 mg GAE/g DW) and the leaves of *A. muricata* (55.18 ± 0.18 mg GAE/g DW). Meanwhile, flavonoid content (TFC) studies revealed that the roots of *A. muricata* have the highest content (317.22 ± 3.47 mg RE/g DW), followed by its bark (201.17 mg RE/g DW) and leaves (181.94 mg RE/g DW). This study discovered that *A. muricata* has the highest TFC, followed by *A. squamosa*, *A. reticulata*, and *A. cherimola* [17]. As a result, this demonstrated that the high amounts of TPC and TFC in *A. muricata* are the reason for its anti-diabetic properties in traditional and medicinal treatment. In this paper, *A. muricata* was chosen to be reviewed for its anti-diabetic effect compared to other *Annona* species because it is the most used in traditional diabetes treatment. This is accomplished by integrating all scientific studies and relating traditional uses to phytochemical content [18].

## 2. *Annona* Plant

### Annona muricata Linn.

**Figure 1:**
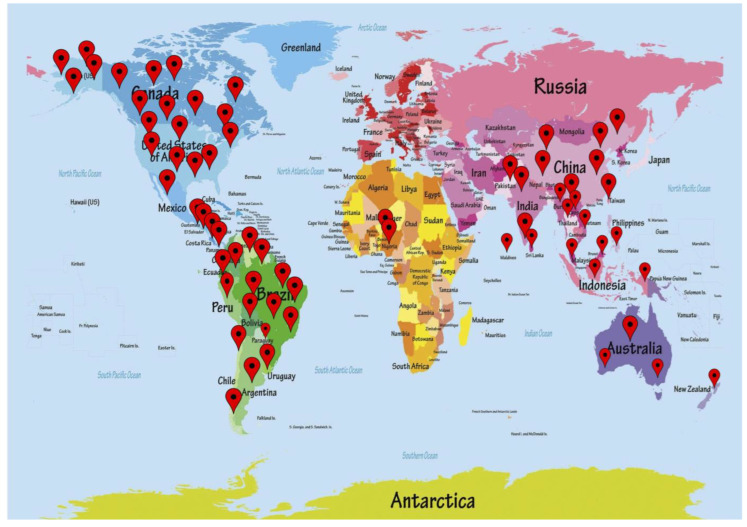
Distribution of *A. muricata* throughout the world.

*A. muricata* L. is recognized as sirsak, graviola, paw-paw, soursop, ‘durian belanda’, and guanabana. *A. muricata* is reported to be cultivated in the warm lowlands of Eastern and Western Africa, Australia, North America, temperate and tropical Asia, the Caribbean, Mesoamerica, and the south-central Pacific Islands. *A. muricata* belongs to the custard-apple genus that contains approximately 125 species. When compared to other Annonaceae family species, this species is the most frequently grown. *A. muricata* is extensively planted for the edible fruits, and it is now naturalized outside of its native ranges in tropical America and Africa, as presented in Figure 1 [19].

**Figure 2:**
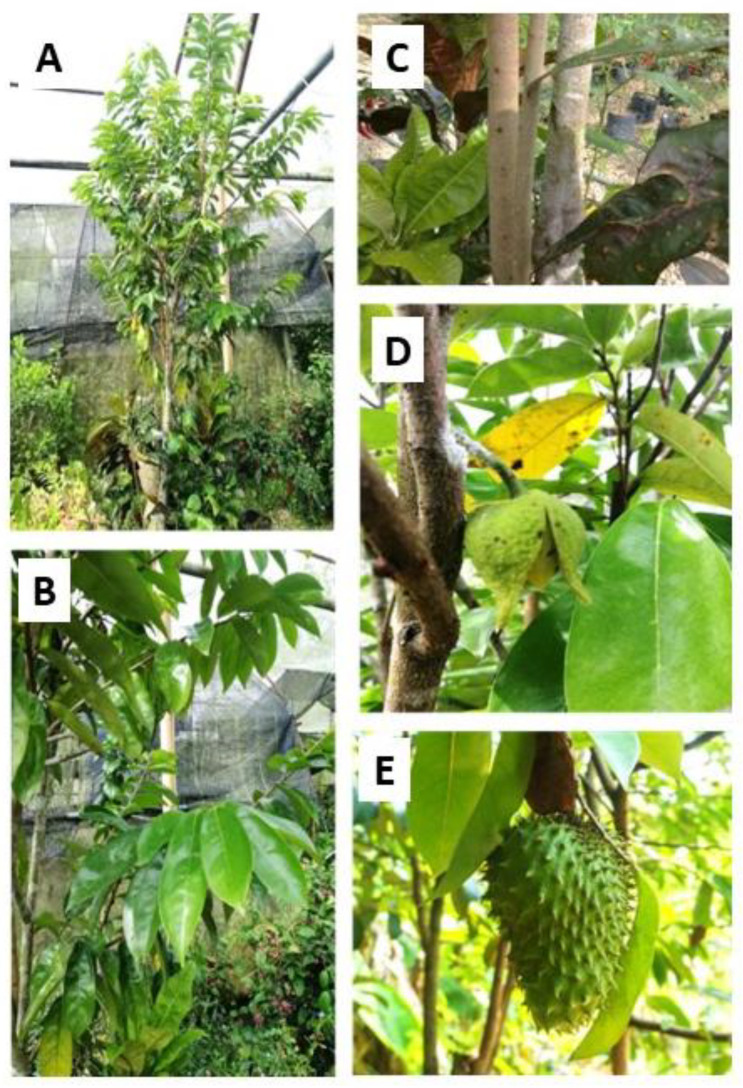
Whole plant (**A**), leaves (**B**), stem (**C**), flower (**D**), and fruit (**E**) of *A. muricata* Linn.

*A. muricata* is a small evergreen tree (Figure 2). It can be slender and upright or low spreading and bushy and becomes straggly as it matures. It is 5 to 10 m tall, 15 to 83 cm in diameter, and has low branches. When the dark-green leaves are crushed, they emit a strong odor [20]. The hermaphrodite flowers, which have a unique fragrance, are generally produced singularly or in small clusters on ancient wood. The flowering phase begins at age 3 to 4, though this may vary depending on the environment. The fruit is heart-shaped to oval, depending on its pollination. The fruiting occurs most of the year, but it may become seasonal, depending on the altitude. The fruit will become distorted with an irregular shape and undersized when it has poor pollination and unfertilized ovules fail to develop. The skin has many recurved soft spines 0.5 to 1.3 cm apart [19]. The fruit’s flesh comprises an edible white pulp, fiber, and a core of indigestible black seeds. The pulp is also used for flavor sweets, sorbets, and ice cream, as well as fruit nectar, smoothies, and fruit-juice beverages. Unless a blender is used for processing, the seeds are usually left in the recipe and removed when eating.

## 3. Ethnomedicinal and Medicinal Uses of *A. muricata*

### 3.1. Ethnomedicinal Uses

**Figure 3:**
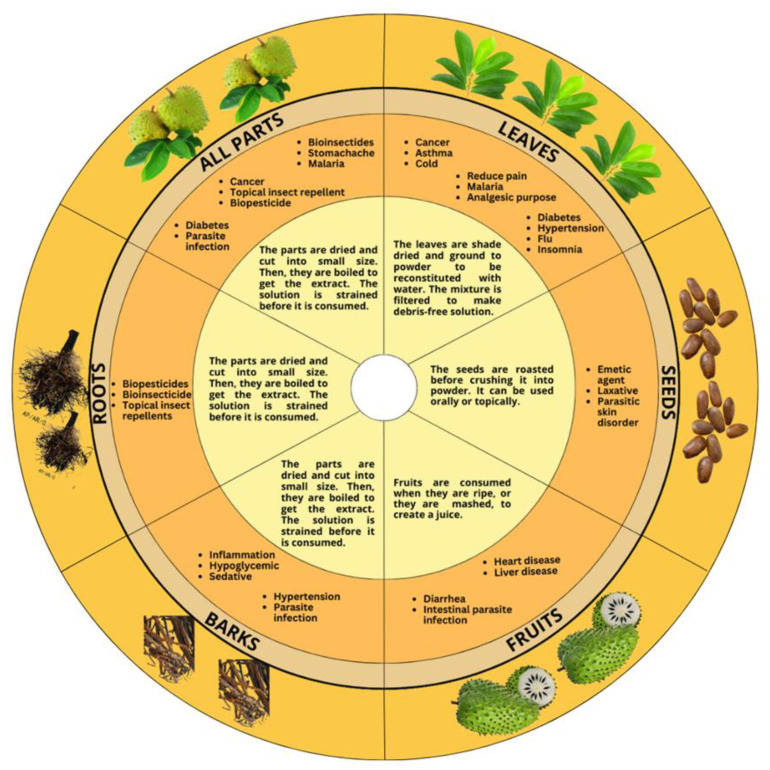
Traditional application and method of preparation for various parts of *A. muricata* Linn.

All parts of *A. muricata* have been used for centuries to cure many ailments and wounds. The method of preparation varies from topical applications, direct ingestion, decoctions, and juicing. Based on a review on the pharmacological activities of *A. muricata*, it has long been used to treat a variety of conditions, including cancer, diabetes, hypertension, respiratory illnesses, fever, and bacterial infections (Figure 3) [21,22]. This plant is widely used traditionally to treat various diseases in South and North America, as well as West Africa [23]. The bark, root, seed, and leaf of *A. muricata* are widely decocted for medical and treatment purposes. In Indonesia and South Pacific countries, soursop leaves are used in a bath to cure skin ailments. Discomfort, such as asthma, colds, and flu, are also managed using the leaves, especially in Mexico and Brazil. Other countries, such as Martinique and Nicaragua, also use it with the same purpose, as stated in a review [24]. Moreover, these leaves are applied topically by people in New Guinea and Ecuador to reduce pain. In Malaysia, the leaves are used to treat external and internal parasite infections and malaria. The leaves are used to treat insomnia, diabetes, headaches, and cystitis [21,22]. In Tanzania, which is in Eastern Africa, the leaves of *A. muricata* are among the most-used plants to cure diabetes. This medication is available locally and is purchased by patients via marketplaces, neighbours, or traditional medicine sellers, and some participants reported that they had cultivated therapeutic plants near their houses. According to local herbalists, they obtained the ingredients they used to treat diabetes locally and created them themselves based on their experience. Its decoction is consumed in minimal doses to lower blood-glucose levels. The interview emphasized that there is a certain quantity that patients must follow since it is highly powerful and may bring adverse effects or an excessive reduction in glucose level. Furthermore, if the medication is in liquid form, most of the patients combine it with warm water, milk, tea, cereal, or drink it directly [21]. At the same time, its decoction is administered topically for anti-rheumatic and neuralgic effects, as well as to reduce abscesses [25]. 

Other than leaves, the fruit is also utilized for medicinal uses. In addition to being tasty, the juice is used to treat heart disease, liver disease, and diarrhoea by acting as a galactagogue. The juice is taken as a beverage in South America to eliminate intestinal parasites. On the other hand, the powder from the toasted seed of *A. muricata* is used as an emetic agent and laxative. Moreover, when mixed with grease to make lotion, the powder can treat parasitic skin disorders [26]. In addition, barks of *A. muricata* are being used to treat hypertension, diabetes, inflammation, parasite infection, and as a smooth-muscle relaxant [2]. In several tropical Sub-Saharan African countries, including Uganda, all parts of *A. muricata* are used to treat malaria, stomachache, parasite infections, diabetes, and cancer. The roots, leaves, seeds, and unripe fruit are also used as bioinsecticides, biopesticides, and insect repellents on the skin. In India, *A. muricata*’s roots, bark, and leaves are reported to have antiphlogistic and anthelmintic effects, while the plant’s flowers and fruits are used to treat catarrh. Furthermore, *A. muricata* aqueous extract is utilized to manage insects, such as lepidopteran larvae, aphids, and thrips [19]. *A. muricata* has been used in combination with other plant species. In Malaysia, a mixture of leaves from *A. muricata*, *Hibiscus rosa-sinensis* L., and *A. squamosa* is made into juice and applied on the head to prevent fainting [19]. In summary, every part of the *A. muricata* tree has its own beneficial effects. Older people also continuously apply this plant in treating certain diseases, showing its effectiveness as a remedy.

### 3.2. Medicinal Uses

*A. muricata* L. is a coveted tropical tree that has been widely used in folk medicine worldwide. Some of these uses are supported by scientific evidence, mostly through in vitro and in vivo studies. Medicinal plants are also protected by stringent intellectual property laws to support their traditional uses in treating various disease conditions. Despite the theoretical differences between traditional and modern medicine, both approaches are complementary. Moreover, *A. muricata* leaves are now utilized to control and treat diabetes, cancer, and hypertension [8]. Acetogenins are the most-abundant phytoconstituents in *A. muricata* and are characteristic of Annonaceae. Other phytochemicals reported in this species are alkaloids and phenolics. Leaves are among the most-researched plant organs, most likely because they are the most commonly utilized as medicine, while the fruits are an exotic commodity. There is mounting evidence that *A. muricata* has anti-cancer properties. This is because of its acetogenin content that has cytotoxic properties, causing apoptosis in cancer cells [2]. Acetogenins are a unique group of derivatives of long-chain fatty acids generated from the polyketide pathway. More than 120 acetogenins have been recorded from the leaves, stems, bark, seeds, pulp, and fruit peel of *A. muricata* in earlier phytochemical studies, and around 46 acetogenins have been discovered from the leaves [19]. 

This plant is commonly used to treat diabetes traditionally and research demonstrated that the leaves and fruit pulp are the most efficient as alternative diabetes treatment. This is partly because this plant has the ability to inhibit α-glucosidase and α-amylase activity, thereby preventing an increase in blood-glucose levels [9]. On the other hand, *A. muricata* leaves are believed to control convulsive seizure and fever, which has been investigated by Gouemo and colleagues. Another study showed that treatment with an ethanolic extract from the leaves reduced the incidence and mortality rate of seizures [2]. Furthermore, leaves, barks, roots, and seeds of *A. muricata* demonstrated high anti-bacterial action against *Staphylococcus aureus*, *Pseudomonas*, *Bacillus, Klebsiella*, and *E. coli,* as suggested by earlier investigations [27]. This anti-bacterial effect may partially clarify the use of leaf extracts to treat urinary tract infections, diarrhea, pneumonia, and skin disease. *A. muricata* has also been used as a biopesticide, especially in controlling mosquito breeding. A study suggested this happened as the plant’s acetogenins can cause toxicity to the mosquito larvae [24]. Furthermore, *A. muricata* may have the potential to boost one’s immune system through the activation of mitogen-activated protein (MAP) kinase signaling pathways [28]. Its phenolic components, which include tannins, flavonoids, phenolic acids, and lignin, can increase the body’s antioxidant potential. This condition reduces inflammation and prevents bleeding and hemorrhoids. Moreover, the ability of *A. muricata* to inhibit α-glucosidase activity better than α-amylase activity, resulting in an anti-diabetic effect, is attributed to its phenolic-rich content [5]. Lastly, based on a review paper, flavonoids and alkaloids in *A. muricata* can decrease blood cholesterols in an animal model, which can lead to a hypotensive effect [29]. 

## 4. Phytochemistry and the Pharmacological Effect of *A. muricata*

Extensive phytochemical analyses on various parts of the *A. muricata* plant have revealed the presence of a variety of phytoconstituents and bioactive compounds. Thus, 212 compounds have been identified in this plant, with acetogenins being the most prevalent [8]. Other compounds that can be identified are alkaloids and phenolics, all of which are listed in Table 1 along with their effects. These compounds were analyzed through High-Performance Liquid Chromatography (HPLC), Nuclear Magnetic Resonance spectroscopy (NMR), Fourier-Transform Infrared spectroscopy (FTIR), Kedde’s reagent, and HPLC coupled with a photodiode array detector (HPLC-DAD) [30,31,32]. 

**Figure 4:**
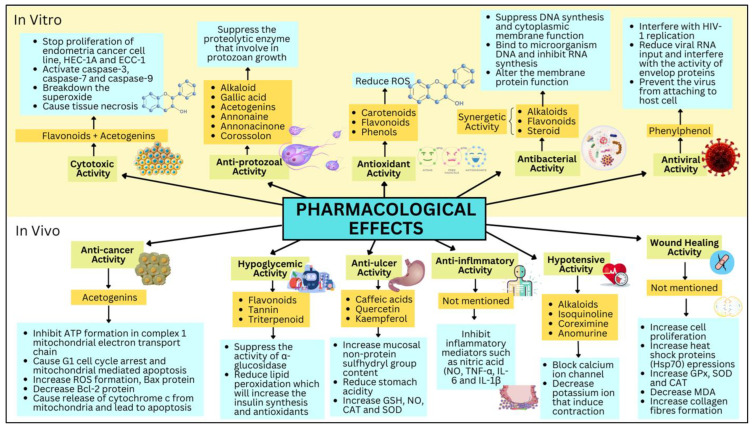
Pharmacological effects of *A. muricata* along with its bioactive compounds and mechanism.

Much research has been carried out on *A. muricata* to evaluate its pharmacological effect, in which a systematic review was conducted to incorporate the scientific studies published up to February 2017, deducing that only 2% had been conducted as clinical trials, 2% in silico modelling, 36% for in vivo studies, and most studies regarding this plant were carried out through in vitro studies [24]. The extract used was mainly based on organic solvents, as opposed to traditional preparations using water. The advantage of solvent extraction is due to the effectiveness in extracting most bioactive compounds [24]. The pharmacological effect of *A. muricata* is simplified in Figure 4, including in vivo and in vitro studies.

### 4.1. In Vitro Studies

#### 4.1.1. Cytotoxic Activity

**Figure 5:**
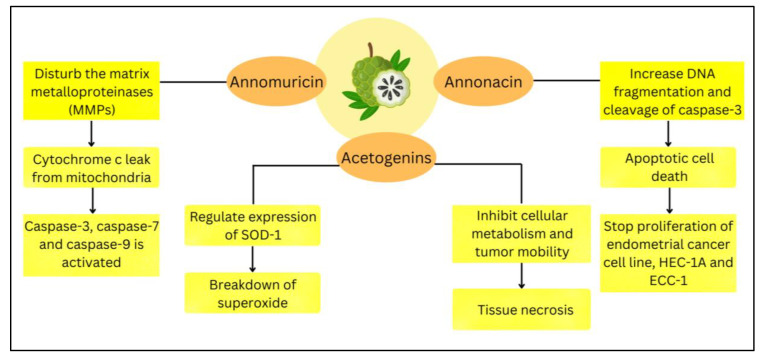
The compounds contained within *A. muricata* and their mechanism to counterattack the cancer cells.

Many investigations have been carried out to study the anti-cancer properties *of A. muricata*. The cytotoxic activity of this plant occurs due to the presence of acetogenin, which is the most-abundant chemical family in various parts of *A. muricata.* The proposed mechanism of action for the selected acetogenins is explained in Figure 5. Acetogenins and flavonoids contained within the leaves can hinder human prostate cancer cell line PC-3 proliferation. This effect occurs as they promote necrosis by inhibiting cellular metabolism and tumor mobility [22]. Annonacin compounds showed the ability to mediate apoptotic cell death by increasing DNA fragmentation and cleavage of caspase-3. This process stops the proliferation of endometrial cancer cell lines, including HEC-1A and ECC-1. The cellular damage can also be prevented using ethanol extract leaves by up-regulating the expression of superoxide dismutase-1 of antioxidant enzyme expression [24]. This expression leads to a breakdown of superoxide, allowing the cell to function. Other than that, annomuricin E is capable of inhibiting HT-29 cell growth. Annomuricin causes cytochrome c to leak from the mitochondria by disturbing the matrix metalloproteinases (MMPs). Thus, pro-apoptotic factors, such as caspase-3, caspase-7, and caspase-9, will be activated [22]. 

On the other hand, the application of *A. muricata* extracts on fibrosarcoma cells (HT1080) can suppress the MMP-2 and MMP-9, hindering cancer progression. The proliferation of human leukemia cell line HL-60 can be suppressed using extracts from *A. muricata* leaves, roots, and twigs [22]. This effect is due to the reduction in reactive oxygen species (ROS) generation, a halt in G0/G1 cell cycle, and a disruption in MMPs. Meanwhile, the administration of ethyl acetate extract and ethanol extract can increase caspase-3 and caspase-9 expression while decreasing Bcl-2 expression. This process activates MCF7 cell apoptosis. Ethyl acetate extract from leaves alone can enhance the expression of caspase-3 in colorectal cancer cell line COLO-205 and breast cancer lines. *A. muricata* extract also has selective action on breast cancer by inducing apoptosis to up-regulate the Bax, down-regulate the expression of Bcl-2, and inhibit the cell cycle at the G0/G1 phase [19]. 

#### 4.1.2. Anti-Protozoal Activity

*A. Muricata* also exhibits therapeutic potential against protozoans that caused amebiasis diseases, chagas, schistosomiasis, malaria, and leishmaniasis [8]. The most-effective part of *A. muricata* in anti-protozoal activity is the seed, as it contains annonacinone, acetogenins, and corossolone [22]. A study showed that the extract from leaves of *A. muricata* can inhibit the growth of *Plasmodium* but is less effective against *Toxoplasma*. This concluded that *A. muricata* has an anti-protozoal effect and the degree of effectiveness varies [80].

#### 4.1.3. Antioxidant Activity

Many diseases (i.e., cardiovascular diseases, arthritis, and cancer) arise due to reactive oxygen species (ROS). Studies showed that *A. muricata* contains vitamins, carotenoids, flavonoids, and phenolic acids, all of which have antioxidant properties (Table 1). The flavonoids, such as gallocatechin, kaempferol, quercetin, rutin, and argentinine, that are abundant in the leaf part may contribute to its potent antioxidant effect and improve other conditions caused by high ROS levels by donating hydrogen [22]. Another study also stated that the ethanolic extract of *A. muricata* is more effective compared to the aqueous extract of the plant since ethanolic extract sustains more secondary metabolites compared to aqueous extract [81]. The content of antioxidant compounds depends on the solvents used for the extraction, in which more compounds can be extracted in polar solvents compared to non-polar solvents [81]. 

#### 4.1.4. Anti-Viral Activity

Regarding anti-viral bioactivity, *A. muricata* extracts exhibit virucidal activity by interfering with HIV-I replication early in the infection. The plant extracts reduce the risk of viral particle transmission by lowering viral RNA input and interfering with the function of envelope proteins during virus entry into the host cell [8]. In addition, it also prevents the virus from attaching to the host cell. The stem and bark of *A. muricata* ethanolic extract showed in vitro anti-viral effects against the herpes simplex virus, in which the minimum inhibitory concentration was 1 mg/mL. Moreover, the acidified ethanolic extract reduced viral multiplication after 1 h of contact. This plant’s anti-viral properties are due to phenolics [22]. It is reported that rutin is the most-abundant component that inhibits viral replication. Furthermore, flavonoid glycosides, quercetin, and naringenin inhibit the spread of SARS-CoV-2 by targeting angiotensin-converting enzymes (ACEs). Meanwhile, studies have shown that quercetin and vanillin have a herpesvirus effect [82]. 

### 4.2. In Vivo Study

#### 4.2.1. Anti-Cancer Activity

**Figure 6:**
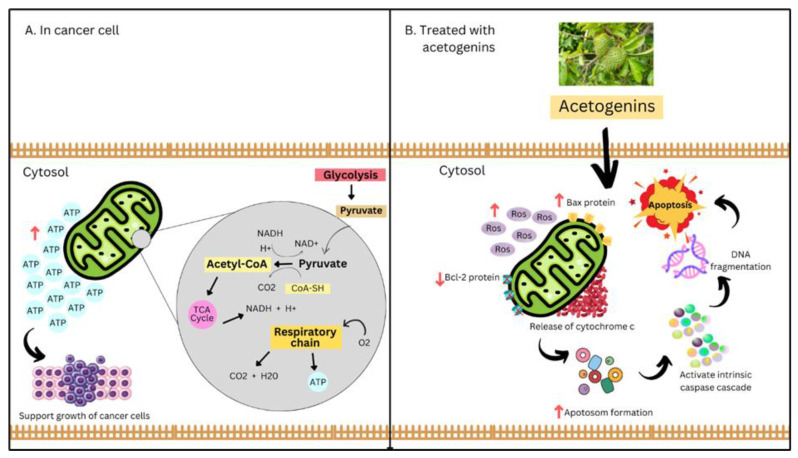
Altered mitochondrial functions in cancer cells and the effect after acetogenin treatment.

A randomized control trial on colorectal patients administrated with 300 mg of *A. muricata* extract containing 0.36% acetogenins after breakfast showed suppression in colorectal cancer cell growth [83]. Acetogenins hamper the ATP formation process required by cancer cells to grow in the complex 1 mitochondrial electron transport chain, as shown in Figure 6. In addition, G1 cell cycle arrest causes mitochondria-mediated apoptosis [22]. Acetogenins induce apoptosis by increasing ROS formation, ann pro-apoptotic Bax protein, and down-regulating antiapoptotic Bcl-2 protein. These processes impair the mitochondrial membrane potential and then cause the release of cytochrome c. This cytochrome c activates apoptosomes and the intrinsic caspase cascade initiates DNA fragmentation, resulting in apoptosis execution (Figure 6). Annocherimolin, an acetogenin, has cytotoxic activity against HT-29 colon cancer cells [24].

On the other hand, the acetogenin compound in *A. muricata* is capable of inhibiting NADH oxidase, which will affect the production of ATP later on. ATP is crucial for cancer cells as it helps them to proliferate [22]. In addition, it also blocks the production of adenosine triphosphate (ADP), which is used by this molecule to activate the pump for cancer drug removal. Hence, acetogenins have been suggested to make chemotherapy more effective. Some studies also proposed that acetogenins have chemotherapeutic potential, especially in cancer cells that have developed resistance to medications [24].

#### 4.2.2. Anti-Ulcer

Gastric ulcers are caused by excessive amounts of gastric acid secreted in the stomach and a decrease in gastric-wall mucus. Moreover, ROS also contributes to this damage. *A. muricata* plants possess gastroprotective properties, most probably due to antioxidant compounds. These compounds can increase the mucosal nonprotein sulfhydryl group content and improve gastric lesions. *A. muricata* extract can reduce stomach acidity and significantly reverse the loss of gastric-wall mucosa, similar to the effects of proton pump inhibitors, such as omeprazole. The *A. muricata* extract improves the amount of several enzymes that can lower cellular ROS, including nitric oxide (NO), glutathione (GHS), catalase (CAT), prostaglandin E2 (PGE-2), superoxide dismutase (SOD), as well as malondialdehyde (MDA) [8]. 

According to a survey, *A. muricata* leaves and bark are frequently brewed as tea to cure digestive issues, such as gastritis and poor digestion. Other preparations of *A. muricata* using ethyl acetate showed anti-ulcer activity by protecting stomach-wall damage and scavenging ROS in rats with ethanol-induced gastric injury. The inhibition of gastric damage is accomplished by up-regulating Hsp70 and down-regulating Bax, which are crucial mechanisms in anti-ulcer action [22].

#### 4.2.3. Anti-Inflammatory Activity

Several studies have shown that *A. muricata* has anti-inflammatory effects, with the leaf being the most commonly studied. *A. muricata* leaf extract inhibits inflammatory mediators, such as nitric oxide (NO), TNF-α, IL-6, and IL-1β; hence, they have the potential to treat inflammation [19]. Oral administration of *A. muricata* ethanolic leaf extracts (10, 30, 100, and 300 mg/kg) significantly reduced carrageenan-induced paw edema, demonstrating the plant’s anti-inflammatory properties. Leukocyte migration and exudate volume were reduced along with this anti-inflammatory action. The same extract, administered orally to mice, significantly reduced abdominal contortions generated by acetic acid (0.6% *v/v*), displaying a potent anti-nociceptive effect [2].

#### 4.2.4. Hypotensive Activity

According to research results, *A. muricata* exhibits hypotensive action, which can reduce blood pressure by blocking calcium ion channels rather than engaging endothelium- and nitric-oxide-dependent mechanisms. Ca+ antagonism during this mechanism tones down the high activity of K+ that can induce contractions [2]. Another study stated that this mechanism did not affect the heart rate but did affect the blood pressure. Administration of *A. muricata* leaf extract to normotensive rats showed significantly declined dose-dependent blood pressure. In addition, the combination of *A. muricata* with *Persea americana* showed a positive result for anti-hypertensive activity [22]. *A. muricata*’s hypotensive effect could be attributed to the alkaloid compounds found in the plant’s leaves. Isoquinoline, coreximine, and anomurine, alkaloids, have been shown to have a transient depressive effect on blood pressure [84].

#### 4.2.5. Wound Healing

This plant showed a compromising wound-healing activity, especially from the leaf and bark extract [2]. A wound heals in four stages: coagulation, inflammation, proliferation, and maturation [8]. Several of these phases are accelerated by the administration of *A. muricata* extract. Heat-shock proteins (Hsp70) expressed during the inflammatory phase are crucial for healing due to their role in cell proliferation, and *A. muricata* caused a significant increase in Hsp70 [2]. A large amount of the cytokines and free radicals produced during this phase by the inflammatory cells might cause lipid peroxidation in the wound. Tissues treated with *A. muricata* extract showed enhanced glutathione peroxidase (GPx), SOD, and CAT activity, which protects tissue from oxidative damage and speeds up the healing process. Furthermore, ethyl acetate leaf extract of *A. muricata* reduces MDA, a lipid peroxidation biomarker that can damage collagen, fibroblast, and endothelial cell metabolism, which are critical for wound healing [85]. This was supported by a study on ethyl acetate extract at a low dose of 5%*w*/*w* and a high dose of 10%*w*/*w* against a wound created on the neck. During the maturation phase, collagen accumulated and fibroblasts multiplied. According to a histological study, *A. muricata* extracts increased the number of collagen fibers deposited in the wound [85].

## 5. Anti-Diabetic Effect of *A. muricata*

DM is a chronic metabolic disorder characterized by high blood-glucose concentrations caused by insulin deficiency, frequently accompanied by insulin resistance. DM is also a leading cause of disability and hospitalization, resulting in a significant financial burden. Many traditional plant treatments for DM are used worldwide. Diabetes management with no side effects remains a challenge for the medical system. As a result, there is a growing demand for natural products with anti-diabetic activity with few side effects [86]. *A. muricata* is known to be traditionally applied in treating diabetes mellitus. Research showed that it has potential bioactive compounds to reduce high blood-glucose levels. Hence, this review emphasizes the anti-diabetic effect of *A. muricata* as well as its pharmacological properties in the hypoglycemic effect. This review aims to aid in strengthening the understanding between traditional medicine, pharmacology, and mechanism of action of *A. muricata* in terms of an anti-diabetic effect. 

### 5.1. Hypoglycemic Activity of A. muricata

Flavonoids in *A. muricata* have anti-diabetic properties. This effect is due to its ability to inhibit the activity of α-glucosidase, as this enzyme is responsible for catalyzing the breakdown of starch into simple sugars. These enzymes help humans digest carbs and starches in their diets to create glucose for intestinal absorption, which raises blood-sugar levels. Hence, this process is inhibited with the presence of flavonoids and prevents the intestine from absorbing the carbohydrate [22]. Moreover, the modulation of glucose absorption, insulin signaling, insulin secretion, and adipose deposition is also supported by flavonoids. They focus on different molecules that are involved in the regulation of several pathways, such as enhancing β-cell proliferation, enhancing insulin secretion, lowering apoptosis, and enhancing hyperglycemia through controlling liver glucose metabolism [87,88]. Some studies linked triterpenoids, tannins, and flavonoids with anti-diabetic activities. These properties may act through various pathways, including promoting insulin production, boosting β-cell repair or proliferation, and amplifying the effects of insulin and adrenalin [86]. 

Aqueous extract of *A. muricata* exhibits anti-diabetic benefits through antioxidant processes. *A. muricata* leaf extract reduced lipid peroxidation processes in streptozotocin-induced diabetic mice and indirectly impacted the synthesis of insulin and endogenous antioxidants [8]. Moreover, studies revealed that diabetic albino Wistar rats had a blood-glucose level reduction after treatment with *A. muricata* extract. Moreover, the pancreatic β cells in diabetic albino Wistar rats also did not exhibit the changes often observed when they were treated with *A. muricata* leaves. Furthermore, the blood-glucose levels, body weight, food and water consumption, lipid profile, and oxidative defenses all returned to normal [15]. 

*A. muricata* fruit extracts have been shown to have antioxidant and anti-diabetic properties in vitro by inhibiting essential type 2 diabetes-related enzymes, such as α-amylase and α-glucosidase. According to a study, its pericarp possesses the highest antioxidant- and enzyme-inhibitory capabilities. Additionally, *A. muricata* seed oil has shown promising anti-diabetic properties against streptozotocin-induced type 1 diabetes. A study revealed that compared to the control group, an experimental mouse treated with *A. muricata* seed oil had considerably lower blood-glucose levels and the pancreatic-islet-preserved area was also improved [22]. In addition to having anti-diabetic properties, according to a review, the extract dramatically decreased serum levels of total cholesterol, low-density lipoprotein, triglycerides, and very-low-density lipoprotein cholesterol [8].

### 5.2. In Vivo and In Vitro Study on Anti-Diabetic Effect

Son et al., 2021, as stated in Table 2, investigated the anti-hyperglycemia-induced liver damage from *A. muricata* extracts in type 2 diabetic mice. After chronically inducing diabetes in C57BL/6J male mice with STZ, two groups of mice were given ethanolic leaves of *A. muricata* extract at 50 mg/kg and 100 mg/kg doses, respectively. Normal and diabetic controls were designated as NC and DMC, respectively, whereas low and high doses of the extract were designated as LAM and HAM, respectively. This study discovered substantial differences in liver weight between rats fed with extract, NC, and DMC [89]. There were no differences in body weight or food consumption between extract-treated and DMC rats (Table 2).

*A. muricata* extract regulated glucose homeostasis by lowering blood-glucose levels. At modest dosages (50 mg/kg of the extract), the fasting blood glucose (FBG) and plasma insulin levels in diabetic rats were significantly decreased. There was a significant difference in haemoglobin A1c (HbA1c) levels and oral glucose tolerance test area-under-the-curve measurement between the LAM and HAM rats and the DMC group. These data supported prior research on the *A. muricata* extract, which reduced blood-glucose levels. A recent study showed that rutin can lower blood glucose and plasma insulin levels, whereas quercetin and kaempferol have hypoglycemic effects through modulating energy balance. The insulin signalling parameter revealed that modest supplementation of ethanolic leaves of *A. muricata* extract improved the insulin signalling pathway via a substantial increase in IRS-1 and GLUT2 proteins compared to the DMC group. As a result, even at modest doses, the injection of an extract comprising rutin, quercetin, kaempferol, and acetogenins appears to be beneficial in controlling diabetes metabolic abnormalities by increasing the insulin signalling pathway [89].

The hepatic morphology and hepatic triglyceride (TG) characteristics of the DMC group had significantly higher fat accumulation than rats in the NC group. Compared to the DMC group, the extract treatment reduced the number and size of lipid droplets. Hepatic TG was abnormally greater in the DMC group compared to the NC group but returned to near normal in the LAM group. All of the effects of the extract on cholesterol demonstrated its capacity to reduce hepatic triglycerides and plasma LDL cholesterol levels, lowering fat buildup in the liver and avoiding NAFLD. AST indicated no changes in hepatic damage. The DMC group, on the other hand, had much greater ALT levels than the NC group. Compared to DMC, rats in the LAM group had significantly lower ALT levels, unlike the HAM group. As a result, treatment of the extract prevents liver injury by reducing fat formation, suggesting that its protective impact was not dose-dependent. *A. muricata* extract is expected to reduce the incidence of diabetic liver disease in the long run while having fewer negative effects on the body [89].

An imbalance of free radicals and antioxidants causes oxidative stress, which harms cells and tissues. The DMC group had considerably higher amounts of oxidation products, 4-HNE, and protein carbonyls than the NC group. Extract therapy, on the other hand, decreased hyperglycemia-induced hepatic oxidative stress. The level of 4-HNE in the HAM group was substantially lower than in the DMC group. Only the amounts of protein carbonyls in the LAM group were considerably lower than in the DMC group. Other oxidative stress variables, including Nrf2 and NQO1 levels, showed significant variations between the DMC and the LAM, showing that extract therapy can ameliorate diabetic tissue damage by reducing oxidative stress [89].

In the energy metabolism indicator parameter, the DMC group had a substantially lower AMPK-mTOR pathway than the NC group. Increased levels of p-AMPK and PGC1 in LAM-treated rats restored energy metabolism. Furthermore, compared to DMC, p-mTOR was considerably lower in the treatment groups. It appears that low doses of extract contribute to the normalisation of energy metabolism by increasing AMPK/PGC1 levels, which are decreased in T2DM. To summarize, a small amount of extracts contain enough quercetin and kaempferol to regulate energy balance and provide a hypoglycemic effect [89].

The autophagy process was suppressed by a reduction in p-AMPK and an elevation in p-mTOR, resulting in reduced LC3-II expression in DMC compared to NC. In contrast, LC3-II expression was considerably higher in extract-treated groups compared to DMC. For lipid metabolism, the proteins associated with fat production in the LAM group were considerably decreased to normal levels compared to DMC in the extract-treated group. As a control, proteins involved in fat synthesis were considerably increased in the DMC group compared to the NC group. Autophagy was restored after a low-extract supplementation by increasing AMPK and decreasing p-mTOR levels. The findings imply that in diabetic conditions, the extract supplementation might regulate hepatic lipid homeostasis, lipophagy activation, and the AMPK-mTOR pathway [89].

In summary, a low dose of extract treatment was more effective in regulating insulin signalling, energy metabolism, and lipid metabolism compared to a high dose of extract. This observation considers that molecular pathways can be selectively regulated at different doses of extract as they contain different amounts of bioactive compounds. Low-dose extract at 50 mg/kg was the most-effective dose and might be tissue-specific in diabetes, with fewer side effects on the body system.

Setiadi et al., 2019, evaluated the anti-hyperglycemic efficacy of an ethanolic extract of soursop leaf (*A. muricata* L.) and acarbose in diabetic rats induced by streptozotocin (STZ). This experimental study involved pre- and post-tests on five groups of rats, two control groups and three treatment groups. The rats are divided into two control groups, (C(-) received water and C(+) received acarbose) and three treatment groups (T1 received 10 mg/BW extract, T2 received 20 mg/BW extract, and T3 received 30 mg/BW extract). The treatment lasted for 1 week, and blood-glucose levels were measured before and after treatment. Blood-glucose levels in the pre-test experimental groups remained the same. Following treatment, all treatment groups showed significant (*p* < 0.05) changes in blood-glucose levels (reduction) after being treated with different doses of leaf extract. Comparing pre- and post-treatment for each group, the paired *T*-test revealed significant differences for the positive control group and treatment groups. Treatment group 3 (T3, 30 mg/kg soursop leaf extract) had the greatest anti-hyperglycemic impact, and it reduced blood glucose similar to the acarbose group (positive control) (Table 2) [90].

*A. muricata* leaves are reported to contain components, such as flavonoids, tannins, and alkaloids, that can heal pancreatic tissue in diabetic patients. *A. muricata* leaves contain flavonoid derivatives, such as quercetin. Flavonoids reduce blood-sugar levels in various ways, including blocking glucose absorption, improving glucose tolerance and uptake by peripheral tissues, boosting insulin production or functioning as insulin, and modulating enzymes involved in carbohydrate metabolism. The flavonoids quercetin and chrysin can serve as hypoglycemic agents at high doses. This prevents an increase in blood-glucose levels by encouraging cells to make more insulin. In vitro studies further reveal that quercetin can impede glucose transport through the intestinal glucose transporters 2 (GLUT2) and 5 (GLUT5), which are responsible for glucose absorption in the small intestine [90]. 

Tannins are known to promote glucose and fat metabolism. They lower blood-sugar levels by boosting glycogenesis. This chemical can also function as an astringent or chelating agent, shrinking the small-intestine epithelial barrier and limiting food absorption. As a result, the rate of blood-glucose levels will be reduced. Tannins can also lower blood-glucose levels by boosting glucose absorption via phosphoinositide 3-kinase and mitogen-activated protein kinase activation (MAPK). Gallotanins and ellagitanins are two types of hydrolyzed tannins reported in *A. muricata*. Gallotanins can improve glucose absorption while preventing adipogenesis. Ellagitanins (insulin-like substance) exhibit characteristics comparable to insulin and can improve glucose transport activity into fat cells in vitro. Furthermore, tannins contain antioxidant properties and have been demonstrated to suppress tumor development [90].

Alkaloids lower blood-glucose levels by blocking glucose absorption in the gut and gluconeogenesis enzymes. They inhibit the enzymes glucose 6-phosphatase and fructose 1,6-bisphosphatase, which decrease the generation of glucose from non-carbohydrate substrates. Alkaloids also boost glucose oxidation via glucose 6-phosphate dehydrogenase, resulting in lower blood-glucose levels [90].

Using alloxan-induced diabetic rats, Sovia et al. (2017) discovered the hypoglycemic and hypolipidemic effects of *A. Muricata* leaf extract. This experimental study involved pre- and post-tests on five groups of rats, two control groups and three treatment groups. Control group Group 1 (G1) was given water and Group 2 (G2) was given alloxan. For the treatment groups, Group 3 (G3) was given 50 mg/extract, Group 4 (G4) was given 100 mg/extract, and Group 5 (G5) was given 200 mg/extract. The treatment was 21 days, and blood-glucose levels were measured before and after treatment. The results revealed that *A. muricata* treatment caused a considerable drop in blood-glucose and cholesterol levels. No changes were detected in the histological structure of the islet of Langerhans [91].

The hypoglycemic impact of flavonoids found in *A. muricata* leaf extract was achieved through boosting insulin production, inhibiting beta-cell death, and regulating proliferation. Flavonoids have also been shown to enhance Ca2+ absorption from isolated islet cells, which drive beta cells to make insulin. As a result, this activity may assist non-insulin-dependent diabetics with no insulin production. The flavonoid also plays a key part in *A. muricata*’s hypolipidemic action since it protects low-density lipoprotein (LDL) from oxidative changes. This hypolipidemic impact is frequently linked to its anti-diabetic and antioxidant properties. *A. muricata*’s anti-diabetic impact lowers the conversion of excess glucose to LDL, while its antioxidant function protects LDL from oxidative damage, lowering the risk of cardiovascular-diabetic problems. A previous study with STZ-induced diabetes showed that *A. muricata* extract may protect and preserve pancreatic cell integrity from oxidative stress, resulting in a considerable improvement in the islet of Langerhans. In alloxan-induced diabetic rats, however, extract treatment did not restore the islet of Langerhans. It might be because the extract dose employed in this study was insufficient to enhance the islet of Langerhans [91].

**Figure 7:**
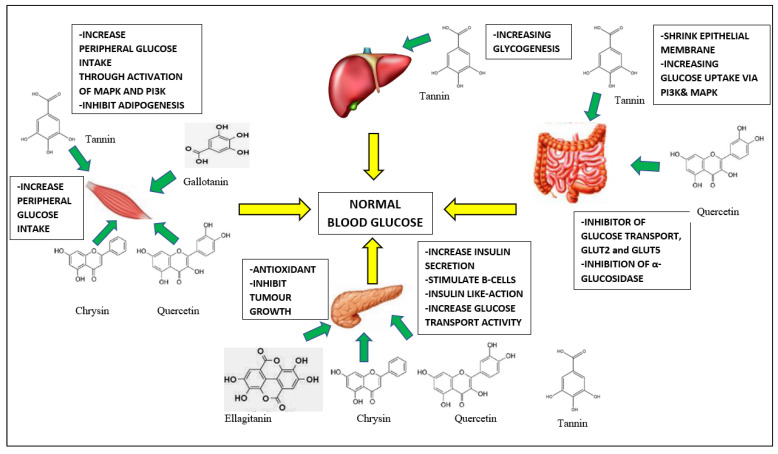
Mechanism of action of *A. muricata* on anti-diabetic effect.

Adefegha et al. (2015) investigated the effects of aqueous extracts (1:100 *w*/*v*) of *A. muricata* fruit parts (pericarp, pulp, and seed) on α-amylase, α-glucosidase, and angiotensin-I converting enzyme (ACE) inhibition in vitro, as stated in Table 2. This study also examined the extracts’ radical scavenging and Fe^2+^ chelation activities, as well as their reducing and phenolic contents [5]. The extracts inhibited α-amylase, α-glucosidase, and ACE activities in a dose-dependent manner. Pericarp extract demonstrated the highest inhibitory effect in α-amylase, α-glucosidase, and ACE from the result of 50% antioxidant activity (EC_50_). In contrast, the seed extract demonstrated the lowest inhibitory activity across all experiments. In all experiments, the inhibitory actions of pericarp extract were lower than those of the positive controls acarbose and captopril [5]. Phenolic distributions in *A. muricata* extracts contributed to their enzyme inhibitory and antioxidant properties. The scavenging of radicals from all extracts confirmed that the pericarp extract had the greatest total phenol and flavonoid content, followed by the pulp and seed, which had the lowest. The inhibition of α-glucosidase was greater than that of α-amylase, which was consistent with a prior study that found plant phenolic-rich extract reduced α-glucosidase activity better than α-amylase activity. Anti-hypertensive ACE inhibitors have been shown to lessen the chance of acquiring type 2 diabetes. The release of bradykinin in response to ACE inhibitor activation may improve the responsiveness of muscle fibres and adipocytes to insulin usage [5]. The ability of the *A. muricata* fruit portions (pericarp, pulp, and seed) to decrease oxidative-stress-induced metabolic illnesses, such as diabetes and hypertension, was highly effective. The mechanism of action of *A. muricata* is simplified in Figure 7.

## 6. Conclusions and Future Perspective

In conclusion, *A. muricata* is widely used in traditional medicine to treat a variety of ailments, such as hypertension, diabetes, and cancer. Research also stated that these plants contain various types of bioactive compounds from certain classes, such as acetogenins, flavonoids, phenols, alkaloids, and megastigmane. In vivo and in vitro research showed that it has potential to treat various conditions, such as wound healing, ulcer, inflammation, cancer, diabetes, and hypertension. In this review, we summarized the traditional uses, medicinal uses, chemical constituents, and pharmacological effects of *A. muricata*. In addition, we also emphasized the effect of *A. muricata* towards an anti-diabetic effect. There is still a significant research gap, and future studies are required to conduct detailed investigations and better understand *A. muricata*’s anti-diabetic potential. In addition, the biological studies conducted by using this crude extract are still limited to anti-diabetic effects. Meanwhile, there is no thorough metabolomic study carried out for this potential effect. The metabolite changes that are revealed from the effect of bioactivities have not been identified. Hence, the mechanisms of action cannot be well determined. Finally, *A. muricata* has a bulk of evidence on the anti-diabetic effect and the potential as an alternative diabetes mellitus treatment. However, major steps to conduct more metabolomic studies to advocate for pharmaceutical development are needed. We also hope that the information offered in this review may encourage clinical studies to be conducted on this potential.

## Figures and Tables

**Table 1:** Bioactive compounds along with analysis tools in different parts of *A. muricata* together with their potential and effects.

Bioactive Compound	Part of Plant	Activity	Effect
Acetogenins			
1. Annomuricin (annomuricin A, B, C) [2,33,34]	Leaves, pericarp	Cytotoxic [33]	Annomuricin A showed toxicity against human breast carcinoma (MCF-7) showed ED_50_ value of >1.0 μg/mL, human colon adenocarcinoma (HT-29) with ED_50_ >1.0 μg/mL, human lung cancer (A549) with ED_50_ of 3.30 × 10^−1^ μg/mL and brine shrimp (BST) LC_50_ result showed value of 6.25 × 10^−1^ μg/mL [33]. On the other hand, annomuricin B revealed toxicity against human breast carcinoma (MCF-7) with ED_50_ value of >1.0 μg/mL, human colon adenocarcinoma (HT-29) with ED_50_ of 4.35 × 10^−1^ μg/mL, human lung cancer (A549) with ED_50_ of 1.59 × 10^−1^ μg/mL and brine shrimp (BST) LC_50_ result showed value of 6.87 × 10^−1^ μg/mL [33]. Moreover, treatment of annonamuricin A, B and C at dose 20 μg/mL reduced PC-3 cell viability by 86.0, 96.9, and 97.7% respectively [34].
2. Annomuricin E [35,36]	Leaves	Cytotoxic [36]	Toxicity towards colon HT-29 cancer cells with ED_50_ values of 6.68 × 10^−2^ μg/mL and pancreatic carcinoma PACA-2 with ED_50_ of 2.42 × 10^−2^ μg/mL [35]. Besides, IC_50_ dose of compounds against HT-29 cells after 12, 24 and 48 hrs of treatment showed a result of 5.72 ± 0.41 μg/mL, 3.49 ± 0.22 μg/mL and 1.62 ± 0.24 μg/mL respectively [36].
3. *Cis*-annomuricin-D-one, *trans*-annomuricin-D-one [37]	Leaves	Cytotoxic [37]	Toxicity against lung cancer cells A549 with ED_50_ of <10^−2^ μg/mL, colon HT-29 with ED_50_ of <10^−2^ μg/mL, and pancreatic PACA-2 with ED_50_ of <10^−2^ μg/mL [37].
4. Annomutacin [38]	Leaves	Cytotoxic [38]	Toxicity against lung A549, breast MCF-7 and colon HT-29 with ED_50_ values of 1.57 × 10^−2^, >1.0 and >1.0 μg/mL respectively [38].
5. Annohexocin [39]	Leaves	Cytotoxic [39]	It showed selective toxicity in lung A549 with ED_50_ of 0.34 μg/mL, pancreatic PACA-2 with ED_50_ of 0.77, colon HT-29 with ED_50_ of 0.78, and breast MCF-7 with ED_50_ of 2.26 μg/mL [39].
6. Annonacin [34]	Seeds, leaves, pericarp	Cytotoxic, insecticidal, anti-microbial, anti-tumor, neurotoxic, neurodegenerative [34]	Anti-proliferative effect on PC-3 cell which the cell viability was decreased by 96.9% with dose at 20 μg/mL [34].
7. *Cis*-annonacin [40]	Seeds	Cytotoxic [40]	Crown gall tumour inhibition (28%), brine shrimp toxicity (LC_50_ of 2.3 μg/mL), lung A549 (LC_50_ of 2.3 μg/mL), breast MCF-7 (IC_50_ of 1.18 μg/mL), and colon HT-29 cancer cell toxicity (IC_50_ of 1.0 × 10^−8^ μg/mL) [40].
8. Annonacin-10-one [41]	Seeds	Cytotoxic, anti-viral [41]	Able to prevent breast cancer activity and halt the SARS-CoV-2 spike protein from relocating [41].
9. *Cis*-annonacin-10-one [40]	Seeds	Cytotoxic [40]	Crown gall tumour inhibition (32%), brine shrimp toxicity (LC_50_ of 1.8 μg/mL), lung A549 (IC_50_ of 3.5 × 10^−1^ μg/mL), breast MCF-7 (IC_50_ of 2.9 × 10^−1^ μg/mL), and colon HT-29 cancer cell toxicity (IC_50_ of 9.0 × 10^−4^ μg/mL) [40].
10. (2,4-cis)-10R-annonacin-A-one, (2,4-trans)-10R-annonacin-A-one [38]	Leaves	Cytotoxic [38]	Toxicity against colon HT-29, breast carcinoma MCF-7 and ling A549 with results of ED_50_ of >1.0, 5.70 × 10^−1^, and 1.74 × 10^−1^ μg/mL respectively [38].
11. Annonacin A [42]	Leaves, seeds, pericarp	Cytotoxic [42]	Could possibly reverse MDR, which is caused by ABCB1 in colorectal cancer. This would make it possible for the tested anti-cancer drugs to work better against tumours [42].
12. Annopentocin A, B, C [37]	Leaves	Cytotoxic [37]	Annopentocin A, B and C have toxicity against lung A549 with ED_50_ of 1.71 × 10^−1^, 2.74 × 10^−2^ and 2.06 × 10^−2^ μg/mL respectively [37]. Besides, these compounds also have effect on colon HT-29 (1.63, 1.64, and 1.24 μg/mL) and pancreatic cancer cells PACA-2 (3.58 × 10^−2^, 1.62 × 10^−1^, and 4.28 × 10^−3^ μg/mL) [37].
13. Annocatalin [43]	Leaves	Cytotoxic [43]	Toxicity in the presence of two types of human hepatoma cells, hep G_2_ and hep 2,2,15 which the IC_50_ result were 5.70 and 3.48 × 10^−3^ μg/mL respectively [43].
14. Annocatacin A [44]	Seeds	Cytotoxic [44]	Toxicity in the presence of hepatoma hep G_2_ and hep 2,2,15 with IC_50_ of 12.11 and 8.17 × 10^−1^ μg/mL respectively [44].
15. Annocatacin B [44]	Leaves	Cytotoxic [44]	Toxicity in the presence of hepatoma hep G_2_ and hep 2,2,15 with IC_50_ of 3.35 × 10^−2^ and 2.22 × 10^−1^ μg/mL respectively [44].
16. Arianacin [40]	Seeds	Cytotoxic [40]	Crown gall tumour inhibition (26%), brine shrimp toxicity (LC_50_ of 7.1 μg/mL), lung A549 (IC_50_ of 4.7 × 10^−3^ μg/mL), breast MCF-7 (IC_50_ of 4.0 × 10^−1^ µg/m), and colon HT-29 cancer cell toxicity (IC_50_ of 4.4 μg/mL) [40].
17. *Cis*-annomontacin [43]	Seeds	Cytotoxic [43]	Toxicity in the presence of two hepatoma cell, hep G_2_ and hep 2,2,15 which the IC_50_ value showed values of 2.98 × 10^−1^ and 1.62 × 10^−2^ μg/mL [43].
18. Annonacinone [45]	Seeds, leaves	Cytotoxic, inhibitory activity, leishmanicidal activity [45,46,47]	Annonacinone enhances the fibrinolytic effect of tissue plasminogen activator (tPA). Annonacinone decreased PAI-1/tPA complex formation by enhancing the substrate pathway [47]. Leishmanicidal activity against *Leishmania* species (*L. donovani, L. major* and *L. mexicana*) which the value of IC_50_, 7.66 ± 0.77, 6.72 ± 0.37, and 8.00 ± 1.00 μg/mL respectively [45]. Moreover, also capable to cause toxicity in lung A549 (126 ± 44 μg/mL), hepatoma hep G_2_ (20 ± 10 μg/mL) and colon Ht-29 (74 ± 7 μg/mL) [46].
19. Annoreticuin-9-one [48]	Seeds	Cytotoxic [48]	Cytotoxic activities against brine shrimp lethality test (BST) with LC_50_ value of 2.4 × 10^–4^ μg/mL, ED_50_ for pancreatic tumour cell line (PACA-2) was 2.4 × 10^–4^ μg/mL; human lung carcinoma (A-549) with ED_50_ = 2.7 × 10^–1^ μg/mL, human prostate cancer (PC-3) showed ED_50_ value of 9.8 × 10^–3^ μg/mL and ED_50_ for human lung carcinoma (A-549) was 2.7 × 10^–1^ μg/mL [48].
20. Asimicin [49]	Leaves	Cytotoxic [49]	Toxicity on HT-29 human colon cancer cell line (ED_50_ of 3.3 × 10^−11^ μg/mL) and lung A549 (10^−3^ μg/mL) [49].
21. Bullatacin [44]	Seeds	Cytotoxic [44]	Toxicity against hepatoma hep G_2_ and hep 2,2,15 with IC_50_ values of 6.30 × 10^−5^ and 6.90 × 10^−5^ μg/mL respectively [44].
22. Bullatalicin [49]	Seeds	Cytotoxic [49]	Toxicity against lung A549 (ED_50_ of 2.34 × 10^−7^ μg/mL), breast MCF-7 (ED_50_ of 2.34 μg/mL), colon HT-29 (ED_50_ 8.8 × 10^−1^ μg/mL), and brine shrimp test BST (LC_50_ of 45.56 μg/mL) [49].
23. *Cis*-annoreticuin [50]	Fruits	Cytotoxic [50]	Toxicity on human hepatoma carcinoma cell line (HepG2) with ED_50_ value of 2.4 × 10^–3^ μg/mL [50].
24. (Sabadelin) chatenaytrienin 1, 2, 3 [51]	Roots	Anti-tumor, cytotoxic [49]	Inhibition of T-Box transcriptional factor (TBX5) and Murine Double Minute 2 (MDM2) [49].
25. Cohibin A, B [52]	Roots, seeds	NR	NR
26. Cohibin C, D [53]	Seeds	NR	NR
27. Corossolone [45]	Leaves, seeds	Cytotoxic [45]	Anti-leishmanial activity with EC_50_ value of between 16.14–18.73 μg/mL [45]. In addition, toxicity against oral KB cancer cells (1 × 10^−1^ μg/mL) [54].
28. *Cis*-corossolone [43]	Leaves	Cytotoxic [43]	Toxicity in the presence of two hepatoma cell, hep G_2_ and hep 2,2,15 which the IC_50_ value showed values of 1.65 × 10^−1^ and 4.76 × 10^−2^ μg/mL [43].
29. Corossolin [54]	Seeds, leaves	Cytotoxic [54]	Cytotoxic activity against oral KB cancer cells (ED_50_ of 3 × 10^−3^ μg/mL) and VERO cells (ED_50_ of 3 × 10^−2^ μg/mL) [54].
30. Corepoxylone [55]	Seeds	NR	NR
31. Donhexocin [56]	Seeds	Anti-tumour, cytotoxic [56]	In vitro inhibition of human leukaemia (HL-60) and human colon adenocarcinoma (HCT-8) cell lines with IC_50_ of <1 μg/mL [56].
32. Gigantetronenin [32]	Leaves, seeds	Cytotoxic, insecticides [32]	Inhibit NADH oxidase (IC_50_ of 3.7 ± 0.1 nM) and cause larva mortility (70% of mortality) [32], toxicity against hep G_2_ (ED_50_ of 7.4 ± 0.1 μg/mL), lung A549 (ED_50_ of 149 ± 7 μg/mL) and MCF-7 cell cancer (ED_50_ of 17 ± 2 μg/mL) [46].
33. Gigantetrocin, gigantetrocin A, gigantetrocin B [49]	Seeds, leaves	Cytotoxic [49]	Gigantetrocin A and gigantetrocin B exert toxicity against colon cancer cells HT-29, ED_50_ of 1.24 and 4.1 × 10^−5^ μg/mL respectively and against lung A549, 3.48 × 10^−3^ and 2.5 × 10^−1^ μg/mL respectively [49].
34. *Cis*-goniothalamicin [40]	Seeds	Cytotoxic [40]	Crown gall cancer cell inhibition (47%), brine shrimp toxicity (LC_50_ of 5.2 μg/mL), lung A549 (IC_50_ of 1.3 × 10^−1^ μg/mL), breast MCF-7 (1.05 μg/mL), and colon HT-29 cancer cell toxicity (5.3 × 10^−3^ μg/mL) [40].
35. (2,4-*cis* and -*trans*) gigantetrocinone [57]	Seeds	Cytotoxic [57]	Toxicity on A549 cell lines (ED_50_ 9.73 × 10^−3^ μg/mL), breast MCF-7 (ED_50_ 2.74 × 10^−2^ μg/mL) and colon HT-29 (ED_50_ 5.49 × 10^−4^ μg/mL) [57].
36. Isoannonacin [49]	Seeds	Cytotoxic [49]	Toxicity against colon HT-29 (ED_50_ 2 × 10^−3^ μg/mL) and lung tumor A549 (ED_50_ 2 × 10^−2^ μg/mL) [49].
37. (2,4-*cis* and -*trans*)-isoannonacin [57]	Seeds, leaves	Cytotoxic [57]	Toxicity against breast MCF-7 (ED_50_ of <10^−3^ μg/mL), lung A-549 (ED_50_ of 4.42 × 10^−5^ μg/mL), and colon HT-29 cell line (ED_50_ of 1.70 × 10^−1^ μg/mL) [57].
38. Isoannonacin-10-one [58]	Seeds	Cytotoxic [58]	Toxicity against colon HT-29 cell line (ED_50_ of 9x10^−3^ μg/mL) and lung A549 (ED_50_ of 7x10^−2^ μg/mL) [58].
39. Javoricin [40]	Seeds	Cytotoxic [40]	Toxicity against brine shrimp (LC_50_ of 4.9 μg/mL), lung A549 (1.7 × 10^−2^ μg/mL), breast MCF-7 (2.3 × 10^−1^ μg/mL), and colon HT-29 cancer cells (1.8 μg/mL), as well as inhibition of crown gall tumours (47%) [40].
40. Longifolicin [59]	Seeds	Cytotoxic [59]	Toxicity in the presence of human hepatoma cells, hep G_2_ (IC_50_ = 4.04 μg/mL) and hep 2,2,15 (IC_50_ = 4.90 × 10^−3^ μg/mL) [59]
41. Muricapentocin [35]	Leaves	Cytotoxic [35]	Toxicity towards pancreatic carcinoma PACA-2 with ED_50_ of 5.03 × 10^−2^ μg/mL and HT-29 colon cancer cells with ED_50_ value of 7.10 × 10^−2^ μg/mL [35].
42. Muricatocin A, B [60]	Leaves	Cytotoxic [60]	Muricatocin A and B showed toxicity against lung cancer cells A549 (ED_50_ of 7.55 × 10^−2^ and 3.34 × 10^−1^ μg/mL), breast MCF-7 (ED_50_ of 1.23 × 10^−1^ and 1.03 × 10^−1^ μg/mL), and colon HT-29 (ED_50_ of 1.56 and 1.66 μg/mL) [60].
43. Muricatocin C [61]	Leaves	Cytotoxic [61]	Toxicity against brine shrimp BST (LC_50_ = 6.04 × 10^−1^ μg/mL), breast cancer cells MCF-7 (ED_50_ = 6.45 × 10^−2^ μg/mL), lung cancer cells A549 (ED_50_ = 9.09 × 10^−2^ μg/mL), and colon cancer cells HT-29 (ED_50_ = 1.48 μg/mL) [61].
44. Muricin H, I [43]	Seeds	Cytotoxic [43]	Toxicity in the presence of two human hepatoma cells, hep G_2_ and hep 2,2,15. Muricin H and I showed IC_50_ of 9.51 × 10^−2^ and 5.09 × 10^−2^ μg/mL against hep G_2_ meanwhile 1.18 × 10^−2^ and 2.22 × 10^−1^ μg/mL against hep 2,2,15 [43].
45. Muricin J, K, L [62]	Fruits	Cytotoxic [62]	Prostate PC-3 cancer cell toxicity [62].
46. Muricoreacin [63]	Leaves	Cytotoxic [63]	Cytotoxicity on colon HT-29 (ED_50_ = 0.57 μg/mL), lung A549 (ED_50_ = 0.23 μg/mL), Breast MCF-7 (ED_50_ = 1.3 μg/mL), kidney A498 (ED_50_ = 0.71 μg/mL) and pancreas PACA-2 (ED_50_ = 2.3 μg/mL) [63].
47. Murihexocin, murihexocin B, C [32,63]	Leaves, pulp	Cytotoxic [63]	Besides, murihexocin B showed larval mortality of 30% [32]. Then, murihexocin C caused toxicity on colon HT-29 (ED_50_ = 1.3 μg/mL), lung A549 (ED_50_ = 1.1 μg/mL), Breast MCF-7 (ED_50_ = 3.8 μg/mL), kidney A498 (ED_50_ = 2.5 μg/mL)and pancreas PACA-2 (ED_50_ = 0.49 μg/mL) [63].
48. Muricadienin [64]	Roots	Cytotoxic [64]	In vitro inhibitory activity against topoisomerases I and IIα, which are key cell cycle enzymes. Then, high cytotoxicity against HEK293 kidney cancer cells (IC_50_ = 0.39 µM) [64].
49. Muridienin 3, 4 [65]	Roots	NR	NR
50. Muricatacin [66]	Seeds	Cytotoxic [66]	Toxicity against lung cancer cells A549 (ED_50_ = 23.3 μg/mL), colon cancer cells HT-29 (ED_50_ = 14.0 μg/mL)and breast cancer cells MCF7 (ED_50_ = 9.8 μg/mL) [66].
51. Muricatetrocin A, B [59]	Seeds, leaves	Cytotoxic [59]	Toxicity against hepatoma cell line, hep G_2_ (IC_50_ = 4.95 × 10^−2^ μg/mL) and hep 2,2,15 (4.83 × 10^−3^ μg/mL) [59].
52. Muricenin [67]	Pulp	Cytotoxic	Toxicity effect against PC-3 cell [67].
53. Murihexol [68]	Seeds	NR	NR
54. Murisolin [54]	Seeds	Cytotoxic [54]	Toxicity against oral cancer KB cell (ED_50_ of 1 × 10^−2^ μg/mL) and VERO cells (ED_50_ of 1 × 10^−1^) [54].
55. *Cis*-reticulatacin [69]	Roots	Anti-protozoal, anti-lymphoma [69]	Inhibition against *Giardia lamblia* (IC_50_ = 59.6 μg/mL) and *Entamoeba histolytica* (IC_50_ = 36.5 μg/mL), anti-lymphoma effect against human lymphoma U937 cell line (EC_50_ = 4.9 ± 0.10 mg/kg) [69].
56. Rolliniastatin 1 [46]	Seeds	Cytotoxic [46]	Toxicity to lung A549 (ED_50_ of >500 μg/mL), hepatoma hep G_2_ (ED_50_ of 12 ± 2 μg/mL), colon HT-29 (ED_50_ of 160 ± 36 μg/mL) and breast MCF-7 (ED_50_ of 17 ± 2 μg/mL) [46].
57. Solamin [54]	Leaves, seeds. roots	Cytotoxic [54]	Toxicity to oral KB cancer cells (3 × 10^−1^ μg/mL) and kidney VERO cells (ED_50_ 1 μg/mL) [54].
58. Squamocin [44]	Seeds	Cytotoxic [44]	Toxicity against hepatoma hep G_2_ (5.47 × 10^−4^ μg/mL) and hep 2,2,15 (9.23 × 10^−4^ μg/mL) [44].
59. *Cis*-squamostatin A, squamostatin D [69]	Seeds	Cytotoxic [69]	*Cis*-squamostatin A and squamostatin D exert toxicity on taxol (resistant cell line) with IC_50_ of 17.40 ± 2.09 μg/mL and 16.19 ± 1.98 μg/mL respectively [69].
60. Xylomaticin [69]	Seeds	Cytotoxic [69]	NR
Alkaloids			
1. Anonaine [70]	Leaves, fruits	Neurotoxic, anti-depressive, anti-plasmodium, dopamine inhibitor, cytotoxic [70]	NR
2. Annonaine [71]	Fruits	Anti-depressive [71]	NR
3. Annonamine [72]	Leaves	Neurotoxicity [72]	Toxicity against neuroblastoma SH-SY5Y (IC_50_ = 195.8 ± 17.0 µM) [72]
4. Asimilobine [71]	Fruits, leaves	Anti-depressive, cytotoxic [71]	NR
5. Coreximine [73]	Leaves, barks, roots, stems	Neurotoxic [73]	NR
6. Isoboldine [74]	Leaves	Anti-malarial [74]	NR
7. Isolaureline [70]	Leaves	Cytotoxic [70]	NR
8. Nornuciferine [71]	Fruits	Anti-depressive [71]	NR
9. (S)-norcorydine [72]	Leaves	Cytotoxic [72]	Toxicity against neuroblastoma SH-SY5Y (IC_50_ = 186.6 ± 29.4 µM) [72]
10. Reticuline [73]	Leaves, barks, roots, stems	Neurotoxic [73]	NR
11. Swainsonine [75]	Leaves, stems	Immune response stimulation [75]	Inhibit lysosomal acidic α-mannosidase to cause neurotoxicity [75]
Phenolic Compounds			
1. Apigenin-6-C-glucoside [76]	Leaves	Antioxidant [76]	NR
2. Argentinine [77]	Leaves	Antioxidant [77]	NR
3. Caffeoylquinic acid [78]	Leaves, pulps	NR	NR
4. Catechin [77]	Leaves	Antioxidant [77]	NR
5. Coumaric acid [79]	Fruits	NR	NR
6. Daidzein [79]	Leaves	NR	NR
7. Emodin [79]	Leaves	NR	NR
8. Epicatechin [77]	Leaves	NR	NR
9. Gallic acid [77]	Leaves	NR	NR
10. Gallocatechin	Leaves	NR	NR
11. Homoorientin [79]	Leaves	Antioxidant [79]	NR
12. Kaempferol [77]	Leaves, pulp	Antioxidant [77]	NR
13. Kaempferol 3-*O*-rutinoside [77]	Leaves, pulp	Antioxidant [77]	NR
14. Robinetin [76]	Leaves	Antioxidant [76]	NR
15. Quercetin [77]	Leaves	Antioxidant [77]	NR
16. Quercetin 3-*O*-glucoside [77]	Leaves	Antioxidant [77]	NR
17. Quercetin 3-*O*-neohesperidoside [77]	Leaves	Antioxidant [77]	NR
18. Quercetin 3-*O*-robinoside [77]	Leaves	Antioxidant [77]	NR
19. Quercetin 3-*O*-rutinoside (Rutin) [77]	Leaves	Antioxidant [77]	NR

NR: Not Reported.

**Table 2:** Anti-diabetic effect of *A. muricata* L. and its bioactive compounds in the involved biochemical parameters determined through in vivo and in vitro studies.

Part of *A. muricata* Extract Used/Test Model/Duration	Dose-Route Administration	Findings of Study	Possible Metabolites	References
• Ethanolic- water (leaves) • C57BL/6J male mice, T2DM (Intraperitoneal (i.p.) injection of streptozotocin (STZ; 60 mg/kg) at once a week for a second consecutive week) • Daily for 9 weeks	Orally gavaged: Group 1: Normal control, NC Group 2: Diabetes mellitus control, DMC Group 3: Low dosage of AME treated group (LAM); DMC + 50 mg/kg of extract Group 4: High dosage of extract treated group (HAM); DMC + 100 mg/kg of extract	(a) Body weight: • No changes between extract treated group and DMC groups (b) Liver Weight: • Significant changes between NC and DMC with extract treated rats (c) Diet Intake: • No changes among all groups (d) FBG and HbA1c: • Rats in LAM & HAM significantly had lower FBG and HbA1c levels compared to the DMC group (e) Glucose intolerance and insulin resistance • Significant difference in blood-glucose levels between extract treated and DMC rats at 30 and 60 min after oral glucose administration (f) Hepatic damage: • Rats in the LAM group has significantly reduced ALT level when compared to DMC, but not for HAM group (g) Hepatic Morphology and Hepatic Triglyceride (TG) and Total Cholesterol (TC): • Lipid droplets and their size were decreased in the AME rats compared to DMC • Hepatic TG reduced to almost normal in LAM rats compared to DMC • No changes for hepatic TC content (h) Insulin Signaling: • Significant difference in IRS-1 and GLUT2 proteins between LAM and the DMC groups (i) Hepatic Oxidative Stress: • A significant difference in levels of 4-HNE, protein carbonyls, Nrf2 and NQO1 between the extract treated rats and DMC (j) Energy Metabolism: • LAM-treated rats have normalized energy metabolism through increased levels of p-AMPK and PGC1α. • p-mTOR was significantly decreased in the treatment groups compared to DMC. (k) Autophagy: • The LC3-II expression level was significantly increased in extract treated groups compared to DMC (l) Lipid metabolism • The proteins related to fat synthesis in LAM treated rats were significantly reduced to normal levels compared to DMC	• Total phenolic • Acetogenins, rutin, quercetin, and kaempferol • Rutin, kaempferol-3-*O*-rutinoside, quercetin, kaempferol, muricoreacin, annonacin, and annonacinone	[89]
• Ethanol extracts (leaves) • 5 groups; 2 control groups and 3 treatment groups [Male white rats (*Rattus norvegicus*). Diabetic rats (STZ-intraperitoneally induced)] • 1 week	(a) Group 1—control (b) Group 2—Acarbose (1.8 mg/kg) (c) Group 3—10 mg/kg (d) Group 3—20 mg/kg (e) Group 3—30 mg/kg	Pre-test: (a) No changes in blood glucose for the pre-test experimental groups Post-test: (a) No changes for the negative control group *p* > 0.05 (b) All treatment groups and the positive control group obtained *p* < 0.05, which means showing significant changes in blood glucose (reduction) The highest anti-hyperglycemic effect was treatment group 3 (30 mg/kg of soursop leaf extract), and it was similar to acarbose group in reducing blood glucose	• Flavonoids (quercetin and chrysin), • Alkaloids • Tannins • Ellagic acid	[90]
• Ethanol extracts (leaves) • 5 groups; 2 control groups and 3 treatment groups [Wistar rats, diabetic rats (alloxan-intraperitoneally induced)]. • 21 days of treatment	Orally gavaged: (a) Group 1—control (b) Group 2—Alloxan treated (c) Group 3—50 mg/kg of extract (d) Group 3—100 mg/kg of extract (e) Group 3—200 mg/kg of extract	• Rats in groups 2 to 5 displayed hyperglycemia after alloxan inducement • Significantly reduce blood glucose for groups treated with the extract • Rats in groups 2 to 5 displayed hypercholesterolemia after alloxan inducement • Significantly reduce hypercholesterolemia for groups treated with the extract • No changes in islet of Langerhans	• Flavonoids • Saponins • Phytosterols • Phenolics	[91]
• Ethanol extracts (fruit) • Groups: 1. α-Amylase Inhibition Assay 2. α-Glucosidase Inhibition Assay 3. Angiotensin-I Converting Enzyme (ACE) Inhibition Assay 4. Free Radical Scavenging Ability 5. Total Antioxidant Power 6. Hydroxyl (OH) Radical Scavenging Assay 7. Determination of Reducing Property 8. Fe^2+^ Chelation Assay 9. Determination of Total Phenol Content 10. Determination of Total Flavonoid Content	In vitro	1. α-Amylase and α-Glucosidase Inhibition Assays: • Extracts inhibited α- amylase, α-Glucosidase and ACE activities in a concentration-dependent manner • The highest inhibition: pericarp extract • The lowest inhibition: seed extract • Extracts showed a lower inhibitory effect when compared to acarbose and captopril in α-amylase, α-glucosidase, and ACE inhibition assay 2. All extracts scavenged DPPH radical in a concentration-dependent manner • The highest DPPH radical scavenging ability: pericarp extract 3. The pericarp extract had the highest scavenging ability of the ABTS radical cation 4. All extracts significantly scavenged OH radical in concentration-dependent manner • The pericarp extract had the highest scavenging ability of the OH free radical • The lowest inhibition: seed extract 5. Extracts chelated Fe^2+^ in concentration-dependent manner • The highest Fe^2+^ chelating ability: pericarp extract • The lowest chelating ability: the seed 6. Pericarp extract had the highest total phenol and flavonoid content followed by the pulp and the seed.	• Phenolics	[5]

## Data Availability

Not applicable.
